# Self-Reported Exposure to ETS (Environmental Tobacco Smoke), Urinary Cotinine, and Oxidative Stress Parameters in Pregnant Women—The Pilot Study

**DOI:** 10.3390/ijerph16091656

**Published:** 2019-05-13

**Authors:** Lubica Argalasova, Ingrid Zitnanova, Diana Vondrova, Monika Dvorakova, Lucia Laubertova, Jana Jurkovicova, Juraj Stofko, Michael Weitzman, Iveta Waczulikova, Martin Simko

**Affiliations:** 1 Institute of Hygiene, Faculty of Medicine, Comenius University, 81499 Bratislava, Slovakia; diana.vondrova@fmed.uniba.sk (D.V.); jana.jurkovicova@fmed.uniba.sk (J.J.); 2 Institute of Medical Chemistry, Biochemistry and Clinical Biochemistry, Faculty of Medicine, Comenius University, 81499 Bratislava, Slovakia; ingrid.zitnanova@fmed.uniba.sk (I.Z.); monika.dvorakova@fmed.uniba.sk (M.D.); lucia.laubertova@fmed.uniba.sk (L.L.); 3 Institute of Physiotherapy, Balneology and Medical Rehabilitation, University of Ss. Cyril and Methodius in Trnava, 91701 Trnava, Slovakia; juraj.stofko@gmail.com; 4 Department of Pediatrics, New York University, New York, NY 10016, USA; Michael.Weitzman@nyulangone.org; 5 Division of Biomedical Physics, Department of Nuclear Physics and Biophysics, Faculty of Mathematics, Physics and Informatics, Comenius University, 81499 Bratislava, Slovakia; iveta.waczulikova@fmph.uniba.sk; 6 IInd Gynecology and Obstetrics Clinic, Faculty of Medicine, Comenius University, 81499 Bratislava, Slovakia; cyklomartin@gmail.com

**Keywords:** environmental tobacco smoke (ETS), pregnant women, questionnaire, urinary cotinine, oxidative stress parameters

## Abstract

Background: Exposure to ETS (environmental tobacco smoke) is one of the most toxic environmental exposures. Objective: To investigate the association of ETS with physiological, biochemical, and psychological indicators, as well as with urine antioxidant capacity (AC) and oxidative damage to lipids in a pilot sample of healthy pregnant women. Methods: Exposure to ETS was investigated via a validated questionnaire, and urine cotinine and the marker of oxidative damage to lipids via 8-isoprostane concentrations using an ELISA kit. Urine AC was determined by the spectrophotometric Trolox-equivalent antioxidant capacity (TEAC) method. From a sample of pregnant women (*n* = 319, average age 30.84 ± 5.09 years) in 80, the levels of cotinine and oxidative stress markers were analyzed. Results: Among the 80 pregnant women, 5% (7.4% confirmed by cotinine) reported being current smokers and 25% reported passive smoking in the household (18.8% confirmed by cotinine). The Kappa was 0.78 for smokers and 0.22 for ETS-exposed nonsmokers. Pregnant women in the ETS-exposed group had significantly reduced AC compared to both the nonsmoker (ETS−) and the smoker groups (*p* < 0.05). Nonsmokers had significantly lower levels of 8-isoprostane than smokers (*p* < 0.01) and ETS-exposed nonsmokers (*p* < 0.05). Correlations between urine levels of cotinine and AC were positive in ETS-exposed nonsmokers. Conclusion: A harmful association of active and passive smoking and oxidative stress parameters among pregnant women has been indicated.

## 1. Introduction

Exposure to environmental risk factors has a negative impact on health, especially in vulnerable population groups, which include the children, mothers, and pregnant women. Exposure to tobacco smoke is one of the most toxic environmental exposures. Environmental tobacco smoke (ETS) causes over 600,000 deaths per year, with more than a third of all people exposed to the harmful effects of smoke. This corresponds to 1% of the global burden of diseases worldwide [1]. Around the world, 40% of children, 33% of male nonsmokers, and 35% of female nonsmokers were exposed to ETS in 2004 [2]. According to the Global Adult Tobacco Survey (GATS) (2008–2010), which investigated the prevalence of smoking and passive smoking among women aged 15–49 years in 14 low- and middle-income countries, the prevalence was 0.4% in Egypt, 30.8% in Russia, 17.8% in Mexico, and 72.3% in Vietnam. In Poland, 26.9% of women smoke, 45.4% are exposed to ETS at home, and 24.3% at work. Slovakia and the Czech Republic did not take part in this survey [3]. According to the WHO, the prevalence of daily adult tobacco smokers in Slovakia in 2016 was 29%, 24% of women and 34% of men [4]. 

Diseases arising from smoking are referred to as “smoking-related diseases”. These include tumors (lips, throat, esophagus, colon, kidney, bladder, liver, lungs), noncancerous respiratory system diseases, cardiovascular diseases, and many other diseases affecting a wide variety of organ systems [5,6,7,8,9,10].

Smoking, however, also affects nonsmokers in households and public places where smoking is allowed [11,12,13]. Nonsmokers are exposed to ETS, a smoke emitted from the burning end of a cigarette or cigar or exhaled by a smoker, which represents a significant health risk [11,12]. Recent studies have demonstrated that ETS is composed of second-hand smoke (SHS) and of third-hand smoke (THS). Third-hand smoke results from residual tobacco smoke pollutants that remain on clothes and hair of smokers and on surfaces, furniture, and dust in indoor environments. Exposure can persist long after smoking has stopped, through the contact with smokers and in indoor spaces in which tobacco is regularly smoked [14,15]. 

There have been many studies pointing to the harmful effects of passive smoking on exposed groups of adults, children, and pregnant women and their fetuses [13,16,17,18,19,20]. The most serious complications of ETS in pregnancy include spontaneous abortion, preterm birth fetal developmental anomalies, ectopic pregnancy, preterm labor, intrauterine growth retardation of the fetus (intrauterine growth retardation or IUGR), fetal death, and sudden infant death syndrome (SIDS) [11,12,21,22,23,24]. Newborns exposed to cigarette smoke during pregnancy have higher rates of neurological disorders with long-term problems in behavioral, emotional, and cognitive functions at later ages [25,26,27]. 

Tobacco smoke contains toxic, carcinogenic, and mutagenic chemicals as well as free radicals and reactive oxygen species with potential oxidative damage to biomolecules. The increased production of reactive oxygen species is related to the depletion of antioxidants and the formation of oxidative stress in the organism [28,29]. As a result, lipid oxidation, cell membrane damage, DNA (deoxyribonucleic acid) strand breaks, and the inactivation of some enzymes may occur [30]. Exposure of pregnant women to tobacco smoke causes oxidative stress not only in pregnant women but also in their fetuses [31,32]. Nicotine and its major metabolite cotinine, with a half-life of about 16 to 20 h (cotinine is the most common biomarker for exposure to cigarette smoke assessed in hair, serum or urine), have high lipid solubility; therefore, they pass rapidly across the placenta into the fetal circulation, with higher levels of cotinine recorded in the fetus than in the mother’s plasma [33,34,35,36,37]. The results of several existing studies indicate that smoking prevalence based on self-report closely approximates estimates assessed by cotinine concentrations. The discrepancy among self-reported smokers and nonsmokers may be due to different smoking patterns, including low nicotine dosing or ETS exposure and misreporting regarding smoking status [34,35,36,37,38].

The aim of this study is to assess the degree of ETS exposure and its association with physiological, biochemical, and psychological indicators and with the urine antioxidant capacity and oxidative damage to lipids in a pilot sample of healthy pregnant women. 

## 2. Materials and Methods

Researchers from the Comenius University’s Obstetrics and Gynecology (OB/Gyn) Department and Institute of Hygiene in Bratislava, Slovakia distributed surveys to pregnant women in the 36th−41st week of pregnancy being seen for the follow-up at the OB/Gyn Department of the Faculty Hospital and Clinic. This survey has been used previously to investigate environmental, behavioral, and psychological factors in the lives of women [20]. The results of that study found that ETS exposure is an independent risk factor associated with worse physical health of nonsmoking mothers and worse mental health among pregnant women [20]. In the present study, we enlarged the sample of pregnant women and validated the self-reported smoking and ETS exposure by the levels of urinary cotinine and assessed selected oxidative stress parameters in urine specimens. The study was approved by the Ethical Committee of the Faculty of Medicine, Comenius University Bratislava, Slovakia and by the Institutional Review Board of New York University School of Medicine, New York, USA (IRB number: 09-0331). 

Exposure to tobacco smoke as well as the analysis of the lifestyle and demographic determinants of passive smoking were assessed by the questionnaire for mothers used in the previous study [18,20]. The survey was based on the MEPS (Medical Expenditure Panel Survey) from the USA [18]. The aim of that study was to compare the physical and mental health of non-smoking mothers living in one household with smokers and nonsmokers. 

Urine specimens were taken at the routine control into plastic containers that were subsequently frozen at −20 °C. In the urine samples, levels of cotinine and oxidative stress marker (8-isoprostanes) were analyzed within 3 months of sampling.

### 2.1. Investigated Population

In the present study, 319 (average age 30.84 ± 5.09) healthy pregnant women without any medical treatment for chronic health conditions were included, and in 80 of them (average age 30.24 ± 4.92 years), the levels of cotinine and oxidative stress markers in urine specimens were analyzed from March to June 2018. The study sample was selected from 55,112 deliveries in Slovakia, 7755 deliveries in Bratislava and 2522 deliveries in the IInd Gynecology and Obstetrics Clinic, Faculty of Medicine, Comenius University Bratislava, Slovakia. The planned sample size was 500 healthy pregnant women. In the sample of healthy pregnant women without any medical treatment for chronic conditions who completed the questionnaire (*n* = 319; average age 30.8 ± 5.09), 79.9% were younger than 35 years old, most of Slovak nationality (94.3%), 78.2% were married or in a relationship, 50.5% graduated from university, 60.6% were employed, and 57.4% had children under 18 years of age in their household (Table 1). Because it would not be feasible to perform the biochemical investigations in the whole sample, a simple random sampling was done. The women were included in a list, each coded with the case-specific ID from the institutional electronic database. This list was then numbered in sequential order from one to the total number of the sample. The sampling procedure was performed in the StatsDirect statistical program utilizing this list as a sampling frame. Assuming estimated minimum sample size of 72 women intended for the biochemical analysis, and anticipated dropout rate of 20%, a total of 100 pregnant women were selected and invited to participate in the study. Of these, 92 replied, and 80 agreed and signed a written consent. The levels of cotinine and oxidative stress markers in urine specimens were analyzed from March to June 2018. In this sample of healthy pregnant women (*n* = 80; average age 30.24 ± 4.92 years), 81.20% were younger than 35 years old, most of Slovak nationality (93.8%), 78.8% were married or in a relationship, 68.80% graduated from the college, 66.3% were employed, and 31.3% had children under 18 years of age in their household (Table 1).

### 2.2. Questionnaire

The “Questionnaire for Mothers” used in our previous studies was administered by a trained person and contained questions on environmental, behavioral, and psychosocial factors in the life of pregnant women [18,20]. In addition to questions on personal (age, nationality, marital status, education, employment, children), behavioral (smoking, lifestyle, nutrition), housing (residence), and economic characteristics (household income), it also included questions on mothers’ smoking and ETS exposure in the household (smoking spouse or other members of the family, number of cigarettes and number of years of smoking). In the case of a former smoker, there was a question for how many years she/he has not smoked. Former smokers were considered nonsmokers.

### 2.3. Chemical Analyses

#### 2.3.1. Cotinine

The level of cotinine was measured in urine samples using a competitive ELISA kit (MyBioSource, San Diego, CA, USA) according to the manufacturer’s instructions. Obtained results were expressed in mg/mol of creatinine. The assay sensitivity was 1 ng/mL.

Pregnant women were assigned into three groups based on urine cotinine levels: 6 women with cotinine levels above 2 mg/mol creatinine were included in the smoker group (S), 15 women with cotinine levels between 0.06–2 mg/mol creatinine into the ETS group (environmental tobacco smoke), and 59 women with cotinine levels below 0.06 mg/mol creatinine were included into the nonsmoker group (NS). 

#### 2.3.2. Antioxidant Capacity of Urine (TEAC)

Trolox-equivalent antioxidant capacity (TEAC) decolorization assay (Re et al., 1999) is a decolorization method applicable for both lipophilic and hydrophilic antioxidants. A cation radical 2,2’-azino-bis-3-ethyl benzothiazoline-6-sulfonic acid (ABTS+) is produced by the oxidation of ABTS with potassium persulfate (K_2_S_2_O_8_). Added antioxidants reduce the cation radical in a dose- and time-response manner. Decolorization of the cation radical is related to the standard trolox (synthetic, water-soluble form of vitamin E). Results are expressed in mmol of trolox/L/mol of creatinine.

#### 2.3.3. 8-Isoprostane

Isoprostane (8-iso prostaglandin F2α) levels in urine were determined by the commercial competitive ELISA kit (Cayman Chemical, Ann Arbor, MI, USA) following manufacturer’s instructions. Results are expressed in ng/mL/mmol of creatinine. Sensitivity of the assay was 3 pg/mL.

#### 2.3.4. Creatinine

Urine creatinine was determined in the certified laboratory (Medirex, a.s., Bratislava, Slovakia).

### 2.4. Statistical Analysis

Descriptive and analytical statistics (categorical data analysis) were employed to identify associations between factors assessed in the questionnaire and self-reported ETS exposure. Kappa statistics, sensitivity, specificity, and correlations were used to determine the extent to which self-reported smoking and exposure to ETS are in agreement with the degree of ETS exposure determined by the levels of urinary cotinine (i.e., to determine the accuracy of self-reported smoking status). Kappa is the percentage of cases in which the two measures are in agreement after accounting for chance agreement [39]. It does not take into account which measure is considered the gold standard. The calculated statistical measures for classification performance of the self-report results included accuracy, sensitivity, specificity, positive predictive value (PPV), and negative predictive value (NPV). Sensitivity is the percentage of true positive (the percentage of respondents who reported being smokers or ETS-exposed nonsmokers among those classified as smokers or ETS-exposed nonsmokers based on cotinine concentrations). Specificity is the percentage of true negatives (the percentage of respondents who reported being nonsmokers among those classified as nonsmokers based on cotinine concentrations). The predictive value positive (PVP) and predictive value negative (PVN) are the complements of the percent false positive and percent false negative, respectively [38,40,41]. Statistical package SPSS, version 24 (International Business Machines Corp., New Orchard Road, Armonk, New York, NY, USA) was used for data analysis. 

To evaluate the results of chemical analysis, the statistical package SPSS version 18 (SPSS Inc., Chicago, IL, USA) was used. The results are expressed as mean ± standard deviation (SD) for normally distributed data, or median (lower quartile–upper quartile) for data not normally distributed. One-way analysis of variance followed with post-hoc Tukey test or its nonparametric alternative and Conover–Iman test were used for the comparison between groups of continuous parameters as appropriate. 

The association between variables was assessed using Spearman and Pearson correlation coefficients. The latter was calculated for mathematically transformed laboratory variables to improve symmetry of their distribution. Multiple linear regression was used to assess explanatory power of multiple independent variables when predicting the cotinine levels.

We used an alpha level of 0.05 as a significance criterion for all statistical tests.

## 3. Results

In the sample of healthy pregnant women in whom we analyzed the levels of cotinine and oxidative stress markers in urine specimens (*n* = 80), there were 5% (4) self-reported smokers, 73.8% (59) self-reported nonsmokers, and 21.2% (17) ex-smokers. The average number of cigarettes was 5.67 ± 4.04 per day, median 5 (2−5); the average duration of smoking was 14.50 ± 7.78 years, median 14.50 (9.0−14.5). ETS exposure (somebody living in the household is smoking) was reported in 25% (20) of nonsmoking respondents. The presence of ETS exposure objectified by cotinine was confirmed in 18.8% (15) respondents. The average number of cigarettes smoked by the partner/person living in the same household was 10.39 ± 6.50 per day, median 10 (5−16.3). The average duration of smoking was 13.43 ± 5.90 years (Table 1). There were 5% (4) self-reported current smokers and 7.4% (6) current smokers as assessed by the level of cotinine in the urine sample and 25% (20) self-reported ETS-exposed nonsmokers and 18.8% (15) ETS-exposed nonsmokers confirmed by the level of cotinine in the urine sample. 

ETS-exposed pregnant women are younger, more of other than Slovak nationalities, are less educated and have fewer children than ETS non-exposed pregnant women. They are from lower-income families and live mostly in rural areas. They eat and live less healthily than women not exposed to ETS. Due to the small sample size, these findings are not statistically significant (Table 2).

The sensitivity for self-reported smoking status was 66.7%, specificity 100%, positive predictive value 100%, and negative predictive value 95.8%. Kappa was 0.78, indicating substantial agreement [42] or excellent agreement [39]. The sensitivity for self-reported ETS exposure was 46.7%, specificity 78%, positive predictive value 35%, negative predictive value 85.2%. Kappa was indicating fair agreement [42] or poor agreement [39]. The agreement for self-reported ETS exposure was better for younger women (≤35 years) and with lower education, reaching moderate or fair to good agreement (Kappa = 0.44) (Table 3) [39,42].

The median value of cotinine in ETS-exposed pregnant women by self-report was 0.22 (0.129–0.338) and in currently smoking pregnant women 253.19 (181.82–498.31) mg/mol creatinine. Pregnant women in the ETS+ group had significantly reduced urine antioxidant capacity (TEAC) compared to both the non-smoker (ETS−) and the smoker groups (Table 4 and Table 5). There was no significant difference in urine antioxidant capacity between the nonsmokers and the smokers. The marker of oxidative damage to lipids—8-isoprostanes were significantly increased in the ETS+ and the smoker group compared to the nonsmoker group. The 8-isoprostane levels were the highest in the smoker’s group; however, there was no statistically significant difference between ETS+ and smokers’ groups.

Significant positive correlation between urine cotinine levels and urine antioxidant capacity (TEAC) in the ETS-exposed group was found (Table 6). 

To investigate the extent to which TEAC, 8-isoprostanes, and questionnaire classification uniquely correlate with cotinine values, multiple linear regression on normalized laboratory variables was performed. Since the stratified correlation analysis suggested some kind of interaction between laboratory values and smoking status (Table 6), interaction terms were also included (Table 7-above). The model confirmed our expectation of high explanatory value of self-reported smoking. Interpretation of self-reported exposure to ETS should be taken with caution due to suspected interaction between the smoking status and TEAC pointing to the possible nonlinear relationship between the variables. Explanatory power of self-reported exposure to ETS could be improved by measuring TEAC (*p* = 0.0994).

## 4. Discussion

The results of our previous studies show that ETS is one of the most important factors associated with the physical and mental health of exposed, nonsmoking partners [20]. That study, using a nationally representative sample from the years 2000 to 2004 in the USA [18], showed a negative relationship between living with smokers and the physical and mental health of nonsmoking mothers with children [18]. The limitation of our previous studies was the fact that the smoking status was ascertained exclusively via self-reporting. Since there is considerable public awareness about the effects of cigarette smoking and ETS exposure, participants might be motivated to underreport their smoking status, although there is evidence in some studies to show that self-report is an accurate way to measure smoking behaviors [38,43,44,45,46,47]. 

The strength of the present study is the determination of the accuracy of self-reported smoking and ETS exposure status by urinary cotinine and investigation of the association of ETS and the urine antioxidant capacity (AC) and oxidative damage to lipids. Active smoking of pregnant women or ETS exposure results in several problems, such as intrauterine growth retardation, an increased risk of spontaneous abortion, reduction of pulmonary function in healthy neonates or a higher risk of sudden infant death syndrome [22]. One of the possible mechanisms explaining these effects is the presence of the smoke-induced oxidative stress leading to the oxidative damage to molecules and to the inflammatory response [48]. Cigarette smoke contains a large number of free radicals as well as metals such as copper, mercury, and zinc [49], which may catalyze the production of very reactive hydroxyl radicals by the Fenton reaction [50]. Smoking may increase oxidative stress not only through the generation of free radicals but also through the depletion of the antioxidant systems protecting the organism against deleterious effects of oxygen radicals. 

In our study, we examined the association of ETS exposure and active smoking on the oxidative damage to lipids and on the antioxidant capacity of urine in pregnant women. In the past decades, numerous studies have shown that 8-isoprostanes are extremely accurate markers of lipid peroxidation [51,52,53,54]. 8-isoprostanes are compounds produced by the non-enzymatic oxidation of arachidonic acid. We have found that pregnant women exposed to ETS had significantly higher oxidative damage to lipids and significantly lower urine antioxidant capacity than nonsmokers not exposed to ETS. These results indicate that ETS-exposed pregnant women are under increased oxidative stress, which is in accord with other studies [55,56,57] Smoking pregnant women had 8-isoprostanes levels similar to the ETS group and antioxidant capacity similar to the nonsmokers. In the smoker group, compared to the ETS and the nonsmoker groups, women are exposed to a higher load of oxidants, which may stimulate compensatory mechanisms leading to an increased antioxidant capacity. Results of other studies on oxidative stress of smoking pregnant women are mixed. Similar results were reported also in plasma and saliva by other studies [28,32,58]. By contrast, Fayol et al. (2005) detected higher plasma antioxidant activity in ETS-exposed pregnant women than in controls [59]. 

In addition, we observed a strong, significant, positive correlation between urine antioxidant capacity and urine cotinine levels only in the ETS+ group. ETS-exposed pregnant women might be sensitive to tobacco smoke and able to correspondingly stimulate their antioxidant system. However, in the smoker group, this correlation was nonsignificantly negative, which might be the consequence of the higher use of antioxidant compounds by the fetus in order to counteract the increased oxidative burden in active smokers.

ETS exposure or active smoking of pregnant women can have negative effects on their fetuses. There are several reports providing evidence of increased oxidative damage to lipids, DNA, and proteins in the blood of such neonates (Kurt et al., 2016) as well as correlations between oxidative stress parameters of pregnant women and their neonates [60]. Increased oxidative damage to important biomolecules in the fetus caused by cigarette smoke has been implicated in the etiopathogenesis of over 100 disorders [59]. Increased consumption of dietary antioxidants might be a potential therapeutic means against increased oxidative stress in ETS-exposed pregnant women and actively smoking pregnant women. 

The validity of self-reported smoking in population surveys remains an important question [44]. In our study, self-report was in strong agreement with validation by cotinine assessment (78% agreement). Sensitivity was 66.7% and specificity 100%. There are studies with higher sensitivities using larger samples [38,44,61,62]. Self-reported nonsmokers who are smokers based on biochemical measurements do not admit their true smoking status [43]. In studies comparing questionnaire responses on smoking status with cotinine measurements, the estimated misclassification rates (self-reported nonsmokers with cotinine levels indicating current smoking) ranged from 0.9% to 9.8% [43,44,62,63]. In our study, the misclassification rate for pregnant smokers was 3.9%.

The agreement for ETS-exposed pregnant nonsmokers is much lower (22% agreement, 46.7% sensitivity and 78% specificity). The misclassification rate for underreported ETS exposure was 10.81%, but for overreported ETS exposure 17.57%. Older and more educated respondents are more likely to overestimate their ETS exposure (Table 4). The pregnancy itself may also play a role in overestimation of ETS exposure. In the analysis of the lifestyle and demographic determinants of passive smoking, ETS-exposed pregnant women are younger, more of other than Slovak nationalities, are less educated, and have fewer children. They are from lower-income families and live mostly in rural areas. They eat and live less healthily than women not exposed to ETS. These results are similar to those of other studies, but due to a small sample size, we cannot state this with a statistical significance [18,20].

The main conclusion of several studies on large population samples is that the validity of self-reported smoking is high in population-based studies, and the use of cotinine measurements for validation purposes may not be necessary [38,43,44]. Further research may assess the optimal cutoff for validating smoking status among specific groups, such as pregnant women. The effects of gender, social conditions, and pregnancy status on the metabolism of nicotine and on smoking behaviors they should be taken into account. Our pilot study adds a new approach, analyzing urinary cotinine together with selected oxidative stress parameters and following their interactions. According to the results reported in this paper, the explanatory power of self-reported exposure to ETS could be improved by measuring TEAC (*p* = 0.0994).

Findings of several studies suggest that most pregnant women disclose their smoking and ETS exposure as well. Universal urinary cotinine screening of pregnant women could help to monitor high risk for adverse pregnancy outcomes [64,65,66]. Pregnancy smoking might fluctuate, as women try repeatedly to quit or cut down. In this case, cotinine measures may be of limited use for validation. They inform only about a recent exposure, vary with individual smoking status, and depend on the time since the last cigarette smoked [61]. More than 15% of rural pregnant women in China with actual exposure to ETS might not perceive themselves as passive smokers in prenatal care, especially in the first trimester [47]. 

Limitations of our study are the small sample size and the cross-sectional design. In our study, we did not use the medical outcomes short form-12 (SF-12) to assess the mental and physical health of pregnant women because pregnancy could influence the mental health (MCS) and physical health PCS scores [20,47,67]. The strength of our study is the separation of current smokers, ETS-exposed and ETS non-exposed nonsmokers and the investigation of the harmful effects of active and passive smoking on detected oxidative stress parameters. 

## 5. Conclusions

Data from our study show that maternal cigarette smoking and ETS exposure during pregnancy may compromise the balance between reactive oxygen species and antioxidant defense and might cause potent oxidative stress with its negative consequences in pregnancy. 

Combining the maternal self-report of smoking with the level of urine cotinine concentration could improve the precision of assessment of exposure to tobacco smoke. Urine testing for cotinine may be useful in reducing the nondisclosure surrounding prenatal tobacco use. This screening could be a valuable tool for counseling to help pregnant women in tobacco smoking cessation. The presented results might be used in clinical practice and in campaigns for smoke-free environments and in the promotion of community-based smoke-free programs. Furthermore, they represent an important argument for intervention in families. A complete smoking ban at home should be considered to avoid potential adverse effects on pregnancy outcomes due to ETS.

## Figures and Tables

**Table 1:** Characteristics of two samples of pregnant women (I (*n* = 319), II (*n* = 80)).

Samples	I		II	
Indicator *	*n*	%	*n*	%
**Age group**				
≤35	255	79.9	65	81.2
>35	64	20.1	15	18.8
**Nationality**				
Slovak	299	94.3	75	93.8
other	18	5.7	5	6.2
**Marital status**				
married/in a relationship	248	78.2	63	78.8
single/divorced	69	21.8	17	21.2
**Number of children under 18**				
no	100	42.6	55	68.7
1	107	45.5	20	25.0
2	24	10.2	5	6.3
≥3	4	1.7	0	0.0
**Mother’s education**				
secondary school or lower	42	13.2	6	7.5
high school graduate	116	36.4	19	23.7
university degree	161	50.5	55	68.8
**Employment status of the mother**				
employed	191	60.6	53	66.3
unemployed	124	39.4	27	33.7
**Father’s education**				
secondary school or lower	63	20.0	10	12.6
high school graduate	118	37.5	21	26.6
university degree	134	42.5	48	60.8
**Employment status of the father**				
employed	307	98.4	78	98.7
unemployed	5	1.6	1	1.3
**Household income**				
≤700 €	62	20.1	5	6.5
>700 €	246	79.9	72	93.5
**Residence**				
urban-metropolitan	229	72.2	55	72.2
rural-non-metropolitan	88	27.8	27	27.8
**Physical activity**				
regular	129	41.1	40	50.6
irregular	185	58.9	39	49.4
**Healthy lifestyle**				
yes	207	65.9	54	67.5
no/not sure	107	34.1	26	32.5
**Number of daily meals**				
≤4	192	60.4	43	54.5
>4	126	39.6	36	45.5
**Smoking status (self-reported)**				
nonsmoker	187	58.6	59	73.8
ex-smoker	103	32.3	17	21.2
current smoker	29	9.1	4	5.0
**Exposure to tobacco smoke (self-reported) ^a^**				
not exposed	156	62.2	56	70.0
exposed	95	37.8	20	25.0
**Smoking status (cotinine objectified—above 2 mg/mol creatinine)**				
no	-	-	74	92.6
yes	-	-	6	7.4
**Exposure to tobacco smoke (cotinine objectified—0.06–2 mg/mol creatinine) ^a^**				
not exposed	-	-	59	73.8
exposed	-	-	15	18.8

* There are some data missing in each variable category. ^a^ If somebody living in the household is smoking.

**Table 2:** Relation between demographic factors and mother’s exposure to tobacco smoke (cotinine objectified—0.06–2 mg/mol creatinine) *n* = 80.

Indicator *	ETS−		ETS+	
	*n*	%	*n*	%
**Age group**				
≤35	10	16.95	4	26.67
>35	49	83.05	11	73.33
**Nationality**				
Slovak	57	96.61	14	93.33
other	2	3.39	1	6.67
**Marital status**				
married/in relationship	41	69.50	11	73.30
single/divorced	18	30.50	4	26.70
**Number of children in household**				
none	40	67.80	12	80.00
1–2	19	32.20	3	20.00
≥3	0	0.00	0	0.00
**Mother’s education**				
secondary school or lower	0	0.00	2	13.33
high school graduate	15	25.42	4	26.67
university degree	44	74.58	9	60.00
**Employment status of mother**				
employed	39	66.10	11	73.33
unemployed	20	33.90	4	26.67
**Father’s education**				
secondary school or lower	5	8.47	3	21.43
high school graduate	16	27.12	3	21.43
college graduate and more	38	64.61	8	57.14
**Employment status of father**				
employed	59	100.00	14	100.00
unemployed	0	0.00	0	0.00
**Household income**				
≤700 €	2	3.4	1	7.10
>700 €	56	96.6	13	92.90
**Urban/rural residence**				
urban–metropolitan area	44	74.58	10	66.67
rural–nonmetropolitan area	15	25.42	5	33.33
**Physical activity**				
regular	35	60.34	4	26.67
irregular	23	39.66	11	73.33
**Number of daily meals**				
≤4	29	49.15	11	78.57
>4	30	50.85	3	21.43
**Healthy lifestyle**				
yes	43	72.88	9	60.00
no/not sure	16	27.12	6	40.00

ETS+ exposed to tobacco smoke; ETS− not exposed to tobacco smoke; * There are some data missing in each variable category.

**Table 3:** Measures of agreement to determine the accuracy of self-reported smoking status and exposure to ETS with the urine cotinine analysis in the sample of pregnant women (*n* = 80).

Measures of Agreement	Smoking Status			
	Nonsmoker vs. Current Smoker	ETS− vs. ETS+		
	Total	Total	Younger Age Group (≤35 Years)	Lower Education
Kappa	0.78	0.22	0.30	0.45
Sensitivity	66.7%	46.7%	54.6%	66.7%
Specificity	100.0%	78.0%	79.6%	80.0%
Positive predictive value	100.0%	35.0%	37.5%	57.1%
Negative predictive value	95.8%	85.2%	88.6%	85.7%
Diagnostic accuracy	96.2%	71.6%	75.0%	76.2%

ETS+ exposed to tobacco smoke; ETS− not exposed to tobacco smoke.

**Table 4:** Cotinine levels, Trolox-equivalent antioxidant capacity (TEAC), and levels of 8-isoprostanes.

Actual Smoking Status	Cotinine	TEAC	8-Isoprostanes
	mg/mol Creatinine	mmol trolox/L/mol Creatinine	ng/mL/mmol Creatinine
ETS− (*n* = 59)	0.00 ± 0.00	1.2 ± 0.4	143.6 (73.91–197.54)
ETS+ (*n* = 15)	0.22 (0.129–0.338)	0.91 ± 0.28	238.41 (112.26–411.88)
current smoker (*n* = 6)	253.19 (181.82–498.31)	1.3 ± 0.43	293.74 (250.17–377.51)

ETS+ exposed to tobacco smoke; ETS− not exposed to tobacco smoke (cotinine validated—0.06–2 mg/mol creatinine for ETS, above 2 mg/mol creatinine for current smokers). Results are expressed as the mean ± SD or the median (lower quartile–upper quartile).

**Table 5:** Statistical significance (*p*-value) of TEAC and levels of 8-isoprostanes.

Smoking Status	TEAC	8-Isoprostanes
ETS+ vs. ETS−	0.0038 *	0.0384 *
ETS+ vs. current smoker	0.0563	0.2614
current smoker vs. ETS−	0.8429	0.0098 *

ETS+ exposed to tobacco smoke; ETS− not exposed to tobacco smoke (cotinine validated—0.06–2 mg/mol creatinine for ETS, above 2 mg/mol creatinine for current smokers); * significant at *p* < 0.05.

**Table 6:** Correlations between cotinine levels in urine and oxidative stress parameters.

Antioxidant Parameters	Rho	*p*-Value
**ETS−**		
TEAC	−0.2036	0.0642
isoprostanes	0.0676	0.3097
**ETS+**		
TEAC	0.7607	0.0007 *
8-isoprostanes	−0.2179	0.2171
**Current smoker**		
TEAC	−0.0857	0.4014
8-isoprostanes	−0.5429	0.1208

ETS+ exposed to tobacco smoke; ETS− not exposed to tobacco smoke (cotinine objectified—0.06–2 mg/mol creatinine for ETS, above 2 mg/mol creatinine for current smokers); Rho—Spearman’s rank correlation coefficient; * significant at *p* < 0.05.

**Table 7:** Multiple linear regression modeling of transformed cotinine levels.

Parameter	Regression Coef.	Partial Correlation Coef.	*p*-Value
Intercept	−2.3265		0.0048
Current smoker_questionnaire (Q)	7.3793	0.8178	<0.0001
ETS+_questionnaire (Q)	0.0712	0.0272	0.8180
ln TEAC (mol Trolox/mol Creatinine)	2.2096	0.4654	<0.0001
ln TEAC * Current smoker_Q	−2.8214	−0.1939	0.0979
ln TEAC * ETS+_Q	−1.5087	−0.1930	0.0994
Ln (Isoprostanes pg/mL/mmol Creatinine)	0.1606	0.1206	0.3062

Analysis of variance from regression: F = 28.97; *p* < 0.0001; multiple correlation coefficient R = 0.8409; R_adj_^2^ = 68.27%. * interactions between the self-reported smoking status, ETS exposure and TEAC; Coef.: coefficient.
